# Engineering Heterostructured Fe-Co-P Arrays for Robust Sodium Storage

**DOI:** 10.3390/ma17071616

**Published:** 2024-04-01

**Authors:** Zidi Xiao, Lin Gao, Shaohui Li

**Affiliations:** 1 Hubei Provincial Collaborative Innovation Center for New Energy Microgrid, College of Electrical Engineering & New Energy, China Three Gorges University, Yichang 443002, China; 15830604367@163.com; 2 Hubei Key Laboratory of Energy Storage and Power Battery, School of Mathematics, Physics and Optoelectronic Engineering, Hubei University of Automotive Technology, Shiyan 442002, China; 3 School of Materials Science and Engineering, Zhengzhou University, Zhengzhou 450001, China

**Keywords:** Fe-Co-P arrays, sodium ion batteries, anode materials, heterostructure, self-supported arrays

## Abstract

Transition metal phosphides attract extensive concerns thanks to their high theoretical capacity in sodium ion batteries (SIBs). Nevertheless, the substantial volume fluctuation of metal phosphides during cycling leads to severe capacity decay, which largely hinders their large-scale deployment. In this regard, heterostructured Fe-Co-P (FeP/Co_2_P) arrays are firstly constructed in this work for SIBs. The novel self-supported construction without insulated binders favors fast charge migration and Na^+^ ion diffusion. In addition, the special heterostructure with abundant heterointerfaces could considerably mitigate the volume change during (de)sodiation and provide increased active sites for Na^+^ ions. Density functional theoretical (DFT) calculations confirm the built-in electric field in the heterointerfaces, which greatly hastens charge transfer and Na^+^ ion transportation, thereafter bringing about enhanced electrochemical performance. Most importantly, the FeP/Co_2_P heterostructure discloses higher electrical conductivity than that of bare FeP and Co_2_P based on the theoretical calculations. As anticipated, the heterostructured Fe-Co-P arrays demonstrate superior performance to that of Fe-P or Co-P anode, delivering high reversible capacities of 634 mAh g^−1^ at 0.2 A g^−1^ and 239 mAh g^−1^ at 1 A g^−1^ after 300 cycles.

## 1. Introduction

To address the energy crisis resulting from limited petroleum storage on Earth, it is significantly crucial to focus on the large-scale conversion and storage of sustainable energy technology. While lithium-ion batteries (LIBs) serve as the primary energy accumulation technology, their limited lithium resources greatly hinder widespread applications [1,2,3,4,5]. Sodium-ion batteries (SIBs) are deemed as a favorable substitute to LIBs thanks to their abundance of raw materials and similar storage mechanism with respect to LIBs [6]. The traditional carbon anodes in SIBs commonly suffer from the problem of limited capacity, resulting in low battery energy density [7]. Although phosphorus occupies the largest capacity of 2593 mAh g^−1^ in SIBs, the large-scale deployment of phosphorus anodes has been extremely retarded by issues such as unsatisfied electrical conduction, enormous volume fluctuation bringing about the cracking of the phosphorus particles, and unsteady solid electrolyte interface (SEI) layer. As a result, the discovery of high-capacity anode materials for SIBs is of significant importance [8]. Recently, there has been extensive interest in transition metal phosphides for SIBs as a consequence of their high theoretical capacity and safe operating potential. Notwithstanding, the substantial volume variation of metal phosphides during cycling easily leads to serious capacity decay, which poses a challenge for their large-scale recruitment [9]. Plenty of strategies have been introduced to settle the abovementioned problems. Constructing nanostructures combined with conductive carbon materials is the commonly used means to modify the electrochemical property of metal phosphides. Engineering nanostructured metal phosphides could greatly reduce the pathway of Na^+^ ions and increase active sites to enhance the reversible capacity. In addition, the incorporation of carbon materials could buffer the volume expansion of host materials and advance electronic conductivity. Nevertheless, the nanostructured metal phosphides inevitably undergo severe agglomeration and nano-cracking during long-term cycling even with the protection of carbon materials. Most importantly, the conventional electrode materials are always mixed with insulated polymer binders to paste on the current collectors, which substantially impedes the charge transfer and deteriorates the electrochemical performance [10,11,12].

Heterostructures have shown significant promise in enhancing electrochemical performance due to the inherent charge transfer driving forces in SIBs [10,11,12,13,14]. In addition, the abundant heterointerfaces within the heterostructures provide sufficient active sites to store Na^+^ ions. However, developing a universally applicable method to produce stable heterostructures still remains challenging [15]. Recently, an innovative FeP/CoP heterostructure has been enveloped in N-doped carbon [16]. This 3D composite features FeP/CoP heterocrystals encased in N-doped carbon, forming a core-shell arrangement. The N-doped carbon’s porous framework establishes a conductive network that propels ion and electron movement, allowing for the cushion of volumetric changes in FeP/CoP and preventing agglomeration while preserving interface integrity. With a well-crafted atomic interface, the FeP/CoP heterostructure not only boosts electronic conduction but also increases active sites to attract additional Na^+^ ions. The FeP/CoP electrode showed a capability of 342 mAh g^−1^ at 5 A g^−1^ and a specific capacity of 525 mAh g^−1^ at 0.2 A g^−1^, coupled with sustained cycling durability exceeding 8000 cycles at elevated current densities. Similarly, CoP/FeP nanoparticles encapsulated in porous carbon nanofibers have been synthesized via coaxial electrospinning followed by a low-temperature phosphorization process [17]. When used as an anode in SIBs, this configuration delivers a high reversible capacity of 459 mAh g^−1^ at 0.05 A g^−1^, maintaining a capacity of 208 mAh g^−1^ at 5 A g^−1^ after 1000 cycles, with a capacity retention of 73.5%. The porous and heterogeneous nature of the CoP/FeP architecture not only guarantees the competitive electrochemical performance but also enables better ion transport and volume expansion accommodation. Concurrently, the strategic design of the heterostructure contributes to an intrinsic electric field that enhances the kinetics reactions [18,19,20].

Nevertheless, there is still no report about the deployment of self-supported bimetallic phosphide Fe-Co-P (FeP/Co_2_P) arrays on Ni conductive networks in SIBs. In this regard, we rationally constructed heterostructured Fe-Co-P arrays in this work. Concurrently, the high theoretical capacities of 926 and 540 mAh g^−1^ for FeP and Co_2_P guarantee high capacity of Fe-Co-P [18,19]. And the special self-supported frameworks without insulated binders greatly boost the electron transfer. Most importantly, the abundant voids within the Ni conductive framework and the Fe-Co-P arrays provide sufficient space for the volume change of the host materials. The special arrays on Ni foam construction allow for the accommodation of the strain of Na^+^ insertion along a specific orientation, leading to enhanced performance. This special heterostructure design effectively mitigates the volume change during (de)sodiation, thereby improving the overall performance of the batteries. In addition, the built-in electric field in the heterointerfaces greatly accelerates charge transfer and Na^+^ ion transportation. Benefited from the state-of-the-art design, the self-supported Fe-Co-P anode demonstrates satisfied electrochemical performance with high reversible capacity of 634 mAh g^−1^ at 0.2 A g^−1^ and acceptable long-term cycling stability (239 mAh g^−1^ at 1 A g^−1^ over 300 cycles).

## 2. Materials and Methods

### 2.1. Preparation of Materials

All chemicals were purchased from Shanghai Macklin Biochemical Company (Shanghai, China) and were used directly without further purification. Ni foam was rinsed with dilute hydrochloric acid and distilled water for subsequent experiments. Initially, a mixture of 2 mmol of Fe(NO_3_)_3_·9H_2_O, 2 mmol Co(NO_3_)_2_·6H_2_O, 6 mmol NH_4_F, and 15 mmol CO(NH_2_)_2_ was dissolved in 60 mL of deionized water and stirred for half an hour. This solution was then placed in an autoclave with Ni foam (3 × 5 cm^2^) and subjected to a hydrothermal reaction at 120 °C for 6 h. For phosphorization, the dried precursor was positioned downstream, with 9.4 mmol NaH_2_PO_2_·H_2_O upstream, and the process was conducted at 350 °C for 2 h under N_2_ to yield Fe-Co-P arrays. Similarly, 2 mmol of Fe(NO_3_)_3_·9H_2_O, 6 mmol NH_4_F, and 15 mmol CO(NH_2_)_2_ were dissolved in 60 mL of deionized water and stirred for half an hour. This solution was then placed in an autoclave with Ni foam, followed by a hydrothermal reaction at 120 °C for 6 h. For phosphorization, the dried precursor was positioned downstream, with 4.7 mmol NaH_2_PO_2_·H_2_O upstream, and the process was conducted at 350 °C for 2 h under N_2_ to yield Fe-P arrays. Amounts of 2 mmol Co(NO_3_)_2_·6H_2_O, 6 mmol NH_4_F, and 15 mmol CO(NH_2_)_2_ were dissolved in 60 mL of deionized water and stirred for half an hour. This solution was then placed in an autoclave with Ni foam, followed by a hydrothermal reaction at 120 °C for 6 h. For phosphorization, the dried precursor was positioned downstream, with 4.7 mmol NaH_2_PO_2_·H_2_O upstream, and the process was conducted at 350 °C for 2 h under N_2_ to produce Co-P arrays.

### 2.2. Characterization and Testing of Materials

The morphology, structure, and composition were analyzed using Cu Kα XRD (Rigaku RINT-2000, Rigaku, Tokyo, Japan, λ = 1.542 Å), FESEM (JSM-6330F, JEOL, Akishima shi, Japan), TEM (JSM-2100, JEOL, Sakishima shi, Japan), and XPS (AXIS Supra, Shimadu, Tokyo, Japan) with monochromatic Al Kα X-ray source (15 kV, 0.834 Å) characterizations. These obtained specimens were served as the working electrode in 2025 coin cells, paired with Na foil counter and reference electrodes. The cell assembling process is in the Ar-filled glove box with H_2_O and O_2_ content less than 0.01 ppm. The cells include a glass fiber (Whatman, Maidstone, UK) separator, with active component mass loading around 2.0 mg cm^−2^. The electrolyte is 1 M NaPF6 in an EC and DMC mixture (1:1 volume). Charge–discharge tests were conducted using LAND CT2001A (LAND Electronic Co. Wuhan, China) in the voltage window of 0.01–3.0 V (vs. Na^+^/Na). Cyclic voltammetry (CV) measurements and alternating current (AC) impedance were conducted on a CHI-660C electrochemical workstation (CHI-660C, Shanghai, China).

### 2.3. DFT Calculations

Density functional theory (DFT) calculations were executed using the CASTEP software (Materials Studio 2018) with a plane-wave basis for electronic wave functions. Ultrasoft pseudopotentials facilitated core and valence electron interactions. GGA-PBE function managed exchange-correlation interactions, with a plane wave cutoff at 381.0 eV. The PBE+U methodology is provided to correct the electronic structure properties of FeP, Co_2_P, and FeP/Co_2_P. A 5 × 5 × 1 Monkhorst–Pack grid was used for Brillouin zone integration on a periodic slab with a three-layered planar substrate and a 15 Å vacuum space to avoid inter-slab interactions. Forces on atoms were minimized to under 0.05 eV Å^−1^ for accurate lattice parameters. The heterostructure layer was modeled using the (001) surfaces of FeP and Co_2_P. 

## 3. Results and Discussions

**Figure 1:**
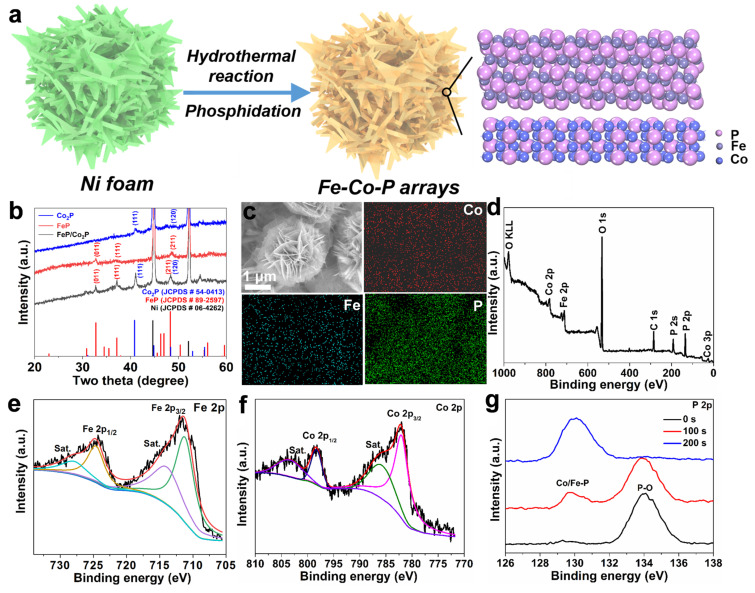
(**a**) Schematic illustration of the preparation process of Fe−Co−P arrays; (**b**) XRD patterns of the three samples; (**c**) SEM and corresponding EDS mapping images and (**d**) the whole XPS survey of Fe−Co−P. High-resolution XPS spectra of (**e**) Fe 2p for Fe−Co−P, (**f**) Co 2p for Fe−Co−P. (**g**) XPS spectra of P 2p for the Fe−Co−P after 0, 100, and 200 s Ar^+^ etching.

Self-supported heterostructured Fe-Co-P arrays on Ni foam were derived via the hydrothermal and followed phosphidation process (Figure 1a). Firstly, Fe(NO_3_)_3_·9H_2_O and Co(NO_3_)_2_·6H_2_O were used as the Fe and Co precursors to grow Fe-Co precursors on Ni foam via the hydrothermal reaction. Subsequently, NaH_2_PO_2_·H_2_O was utilized as the phosphorus source to convert Fe-Co precursors to Fe-Co-P arrays on Ni foam. The NaH_2_PO_2_·H_2_O will begin to decompose and release PH_3_ when the temperature is above 300 °C. The phosphorization temperature at 350 °C is also based on the previous works [9,16]. The gap between FeP and Co_2_P crystals in the Fe-Co-P structure represents grain boundaries and heterointerfaces, which favor the sodium ion storage capability. XRD patterns of the derived samples (Figure 1b) can be well indexed to the coexistence of FeP (JCPDS No. 89-2597) and Co_2_P (JCPDS No. 54-0413), in addition to the diffraction peaks of Ni substrate [12]. For the FeP arrays, the peaks at 30.7°, 37.5°, 48.2° correspond to the (011), (1 11), and (211) planes of orthorhombic FeP with Pnma space group; the peaks at 40.9° and 48.8° correspond to (111) and (120) planes of hexagonal Co_2_P with P62m space group, demonstrating the successful fabrication of FeP/Co_2_P, FeP, and Co_2_P arrays on Ni foam. The SEM image in Figure 1c depicts the typical nanosheet-assembled microspheres with diameter of 2 µm and the parallel elemental mappings reflect the Fe-Co-P arrays grown on Ni foam [13]. It is noted that the nanosheet-assembled microspheres are conductive to the thorough permeation of electrolyte into the inner construction of the electrode materials and robust Na storage capability. In addition, the novel porous construction could largely buffer the volume change and guarantee the structural integrity even under rapid (de)sodiation [21]. XPS characterization is introduced to further investigate Fe-Co-P arrays. The whole XPS survey shows the existence of Fe, Co, P, and O elements in the Fe-Co-P arrays (Figure 1d). The presence of O could be attributed to the adsorbed O_2_ when the Fe-Co-P arrays were exposed in air before the test [14]. The similar phenomena of strong oxygen-related peak are also observed in other related literatures [22,23,24]. In the Fe 2p spectrum of FeP/Co_2_P (Figure 1e), the peaks centered at 711.2 and 724.6 eV correspond to Fe 2p_3/2_ and Fe 2p_1/2_ orbitals, along with two satellite peaks located at 713.9 and 727.9 eV [16]. The high-definition Co 2p XPS spectrum can be classified into four peaks at 803.8, 798.4, 786.3, and 710.9 eV (Figure 1f). The two peaks at 803.8 and 786.3 eV can be explained by the Co 2p_1/2_ and Co 2p_3/2_. The other two signals at 798.4 and 710.9 eV are related with the satellite peaks. In the high-resolution P 2p XPS spectrum for the Fe-Co-P after 0, 100, and 200 s Ar^+^ etching (Figure 1g), the signals at 129.4 eV and 130.5 eV comply with the binding energies of P 2p_3/2_ and P 2p_1/2_ in Fe-Co-P, whereas the peak at 134.6 eV is associated with the surface oxidation of Fe-Co-P [22]. After the Ar^+^ etching for 200 s, the peak for the Co/Fe-P bonds becomes obvious and the peak for P-O bond disappears, illustrating the successful obtainment of Fe-Co-P arrays.

**Figure 2:**
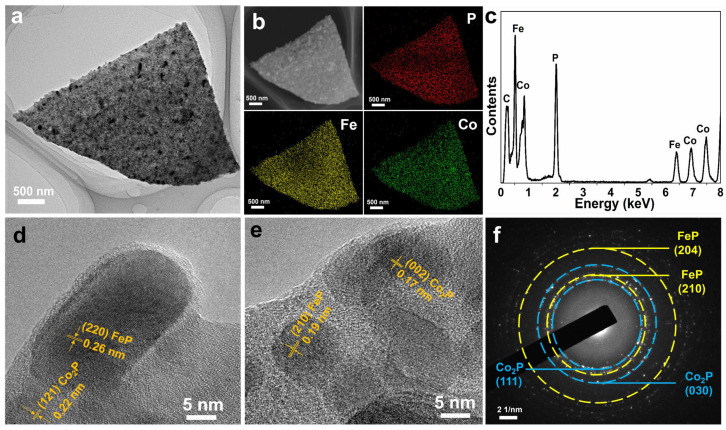
(**a**) TEM image of Fe−Co−P and (**b**) the corresponding element mappings of P, Fe, and Co. (**c**) EDX spectrum, (**d**,**e**) HRTEM image, and (**f**) SAED pattern of Fe−Co−P.

The TEM technique was adopted to observe the inner structure of the Fe-Co-P (Figure 2). The individual Fe-Co-P nanosheet with length around 2 µm can be obviously seen in the view (Figure 2a). The correlated STEM-EDS elemental mappings confirm the existence of Fe, Co, and P elements in Fe-Co-P nanosheet (Figure 2b) [25,26]. The energy dispersive X-ray (EDX) spectrum further reveals the existence of Fe, Co, and P in Fe-Co-P arrays (Figure 2c). The visible lattice spacings of 0.26 and 0.22 nm are related to the (220) facet of FeP and (121) plane of Co_2_P in the HRTEM image (Figure 2d), elaborating the formation of FeP/Co_2_P heterostructures. Lattice spacings of 0.17 and 0.19 nm related to the (002) facet of Co_2_P and (210) plane of FeP (Figure 2e), and the selected area electron diffraction pattern (Figure 2f) clarifies the formation of FeP/Co_2_P heterostructures again.

**Figure 3:**
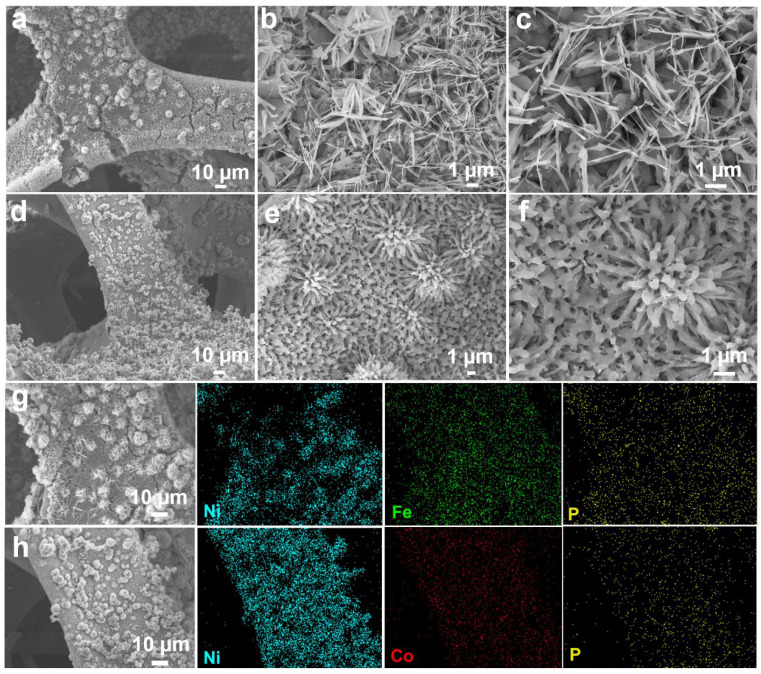
SEM images of (**a**–**c**) Fe−P and (**d**–**f**) Co−P arrays under different magnifications. EDS elemental mappings of (**g**) Ni, Fe, and P in Fe−P arrays as well as (**h**) Ni, Co, and P in Co−P arrays.

**Figure 4:**
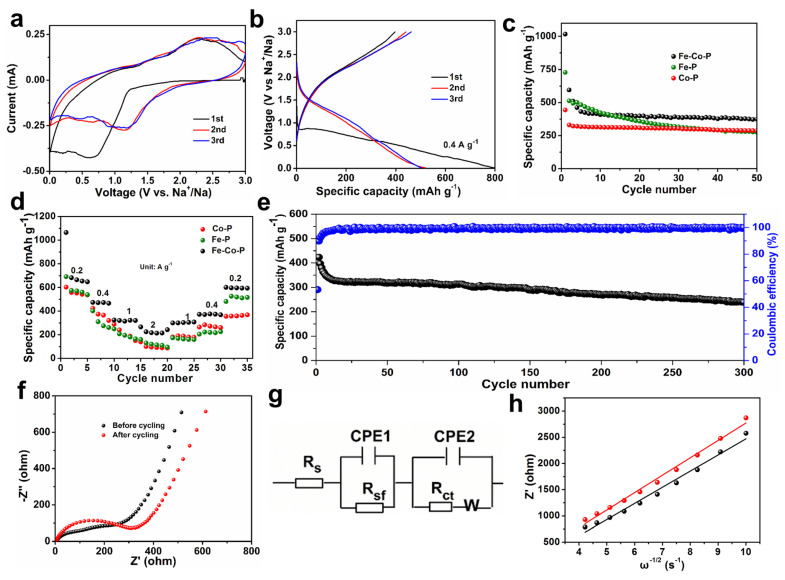
(**a**) CV curves of Fe−Co−P anode at 0.2 mV s^−1^. (**b**) Charge–discharge curves of Fe−Co−P anode at 0.4 A g^−1^. (**c**) Cycling performance of the three electrodes at 0.4 A g^−1^. (**d**) Rate performance of the three electrodes at different current densities. (**e**) Cycling performance at 1 A g^−1^ for the Fe−Co−P anode. (**f**) EIS spectra of Fe−Co−P anode before and after cycling. (**g**) EIS fitting circuit. (**h**) The relationship between low frequency and real resistance for Fe−Co−P anode before and after cycling.

In addition, SEM images of Fe-P and Co-P arrays are also provided in Figure 3a–f with different magnifications. Interestingly, the Fe-P sample shows a typical nanosheet morphology, while Co-P specimen reveals a nanoflower characterization. Combined with EDS elemental mappings, it can be concluded that the Fe-P and Co-P arrays are uniformly distributed on Ni substrate (Figure 3g,h). Half cells were packaged to examine the electrochemical behaviors of Fe-Co-P, Fe-P, and Co-P anodes in SIBs. Cyclic voltammetry (CV) sweeps of the Fe-Co-P anode in the initial three cycles are supplied in Figure 4a to detect the redox reactions upon (de)sodiation. During the first cathodic process, the broader reduction peak at 0.61 V contains two processes, one for the formation of the SEI layer and one for the conversion reaction process of FeP to Fe and Na_3_P and Co_2_P to Co and Na_3_P [27,28,29,30,31,32,33]. The oxidation peak at 2.27 V is predominantly caused by the desodiation course. The cathodic peaks at 1.14 V in the subsequent two cycles disclose a structural progression in the preliminary sodiation stage [34,35]. The overlapped redox peaks in the succeeding two loops indicate the superb reversibility of the Fe-Co-P electrode [36,37,38,39,40]. During the first cycle, the active materials may undergo activation processes such as phase transformation, formation of SEI layer, and changes in electrode morphology. These activation processes can lead to different electrochemical behaviors in the first cycle compared to subsequent cycles. Concurrently, the interface between the electrode and electrolyte may undergo changes during cycling, leading to differences in the electrochemical behavior. These changes can be attributed to side reactions, dissolution of active materials, and other factors that can affect the overall performance of the battery. Overall, the differences in the shape of the CV and charge/discharge curves between the first cycle and subsequent cycles in a battery system are mainly due to a combination of factors including activation of active materials, formation of SEI layer, and changes in the electrode/electrolyte interface. Charge–discharge plots of the Fe-Co-P anode at 0.4 A g^−1^ in Figure 4b are identical with its CV result. Cycling performance of Fe-Co-P, Fe-P, and Co-P anodes at 0.4 A g^−1^ with capacities of 375, 276, and 288 mAh g^−1^ remained over 50 cycles (Figure 4c), demonstrating the most excellent electrochemical performance of Fe-Co-P anode and concurrently illustrating that the construction of FeP/Co_2_P heterostructure largely enhances the reversible capacity relative to bare Fe-P and Co-P anodes.

The cycling stability of Fe-Co-P shows better cycling performance relative to traditional powders owing to the special array construction, which allows for the accommodation of the strain of Na^+^ insertion along a specific orientation [4]. The enhanced capacity of Fe-Co-P mainly comes from the additive active sites in the heterointerfaces. Rate performance of the three anodes is provided in Figure 4d, in which the Fe-Co-P anode exhibits the highest capacities of 634, 467, 328, 213 mAh g^−1^ at 0.2, 0.4, 1, and 2 A g^−1^. When the current density returns to 0.2 A g^−1^, a reversible capacity of 594 mAh g^−1^ can be still achieved. In comparison, the Fe-P anode manifests inferior rate capability with capacities of 571, 310, 195, and 120 mAh g^−1^ at 0.2, 0.4, 1, and 2 A g^−1^. Similarly, the Co-P anode also reflects unsatisfied performance with capacities of 558, 375, 196, and 96 mAh g^−1^ at 0.2, 0.4, 1, and 2 A g^−1^. It is noted that the Fe-Co-P anode delivers a reversible capacity of 239 mAh g^−1^ over 300 cycles with Coulombic efficiency close to 100% (Figure 4e). The relatively low initial Coulombic efficiency and capacity decay in the initial state for the Fe-Co-P anode during cycling could be ascribed to the side reaction between electrode materials and electrolyte together with the gradual electrolyte infiltration into the interconnected construction in the initial step. The performance of the Fe-Co-P anode is better than that of the previous works [5,9,17,18]. The excellent cycling stability of Fe-Co-P is owing to the special array construction, which allows for the accommodation of the strain of Na^+^ insertion [4]. To further clarify the exceptional property of the Fe-Co-P arrays, we conducted electrochemical impedance spectroscopy (EIS) before and after 300 cycles (Figure 4f). The equivalent circuit diagrams are shown in Figure 4g. *R*_s_ represents the electrolyte resistance, while *R*_sf_ is associated with the resistance of the SEI film and *R*_ct_ is related to the charge-transfer resistance. Commonly, the values of *R*_s_ and *R*_sf_ relative to *R*_ct_ can be omitted. The *R*_ct_ values for the Fe-Co-P electrode were estimated to be 255.3 and 311.3 Ω before and after 300 cycles, disclosing the stable inner resistance of Fe-Co-P electrode during cycling. Furthermore, the diffusion coefficient of sodium ions (*D*) can be estimated using the subsequent equations:

*Z^′^* = *R*_s_ + *R*_sf_ + *R*_ct_ + *σω*^−1/2^ (1)

*D* = *R*^2^*T*^2^/2*A*^2^*n*^4^*F*^4^*C*^2^σ^2^ (2)

where *R*, *T*, *A*, *n*, *F,* and *C* denote gas constant (8.314 J K^−1^·mol^−1^), room temperature (298.15 K), electrode surface area (1.539 cm^2^), the number of electrons per molecule that transfer (n = 1), Faraday constant (96,485 C mol^−1^), and sodium ion concentration (1 × 10^−3^ mol cm^−3^). The *D* values of the Fe-Co-P electrode before and after 300 cycles were estimated to be 1.58 × 10^−13^ and 1.36 × 10^−13^ cm^2^ s^−1^ (Figure 4g,h), with no obvious change. Based on the results of AC (alternating current) impedance and diffusion coefficient of sodium ions, it is noted that the Fe-Co-P electrode manifests the most impressive electrochemical stability even underneath repetitive (de)sodiation [39,40,41]. It is noted that the special FeP/Co_2_P heterostructure endows the Fe-Co-P arrays with built-in electric field as well as accelerated charge transfer and Na^+^ ion transportation. Furthermore, the special FeP/Co_2_P heterostructure also plays an important role in mitigating the volume change that occurs within the host materials during the charge–discharge stage [42,43,44,45]. 

**Figure 5:**
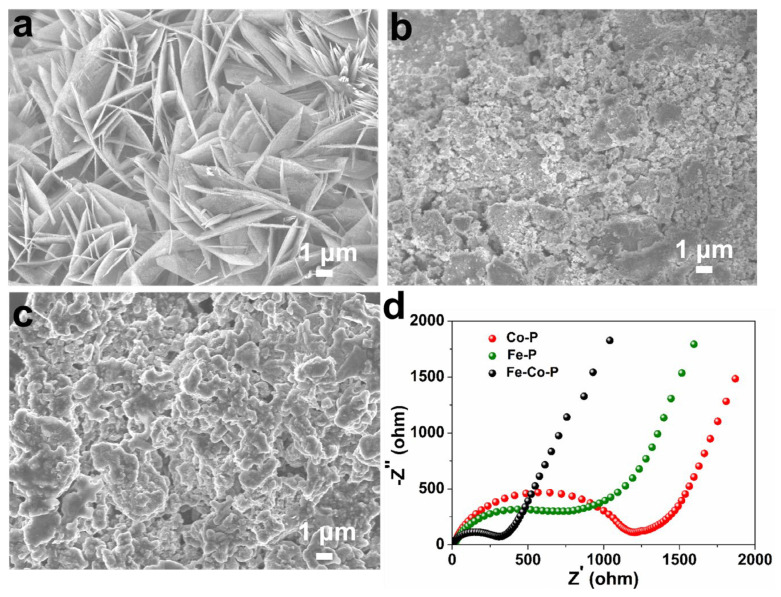
SEM images of (**a**) Fe−Co−P, (**b**) Fe−P, and (**c**) Co−P arrays undergoing 50 cycles at 0.4 A g^−1^. (**d**) EIS spectra of the three electrodes after 50 cycles.

SEM images of the three specimens after 50 cycles at 0.4 A g^−1^ are provided in Figure 5a–c. It is noted that the Fe-Co-P still maintains the nanosheet array morphology undergoing repeated charge–discharge process, while for the Fe-P and Co-P arrays, an obvious agglomeration and pulverization phenomenon is detected. This result suggests that the Fe-Co-P arrays reflect the most robust construction, which might be due to the novel FeP/Co_2_P heterostructured design. The abundant interfaces between FeP and Co_2_P not only provide additive sites to store Na^+^ ions but also effectively buffer the volume change of metal phosphides during cycling, leading to improved construction stability. In addition, the EIS spectra of the three electrodes after 50 cycles are displayed in Figure 5d. The Fe-Co-P anode still shows the lowest inner resistance based on the EIS result.

**Figure 6:**
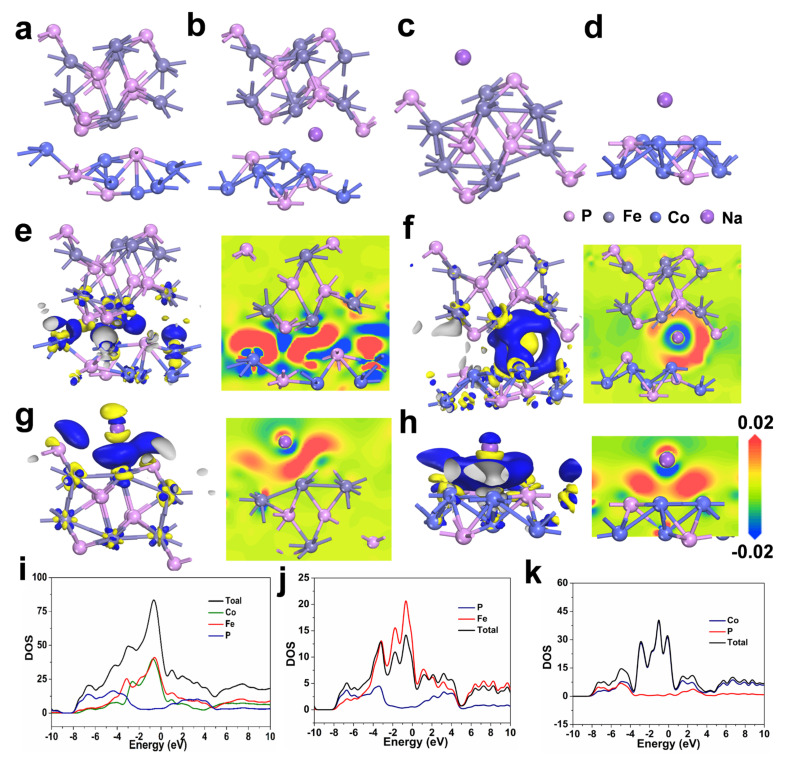
The optimized crystal constructions of (**a**) Fe−Co−P, Na adsorbed (**b**) in Fe−Co−P, (**c**) FeP, and (**d**) Co_2_P. 3D and 2D slice charge density difference of (**e**) FeP/Co_2_P. 3D and 2D slice charge density difference of (**f**) Na adsorbed in Fe−Co−P, (**g**) FeP, and (**h**) Co_2_P. Density of states of (**i**) Fe−Co−P, (**j**) FeP, and (**k**) Co_2_P.

Density functional theory (DFT) calculations were further introduced to deeply probe the underlying mechanism for the excellent performance of FeP/Co_2_P heterostructure in SIBs. The optimized crystal constructions of Fe-Co-P, Na adsorbed in Fe-Co-P, FeP, and Co_2_P are displayed in Figure 6a–d. The charge density difference of FeP/Co_2_P in Figure 6e demonstrates electron transfer from FeP to Co_2_P, which generates an intrinsic electric field at the interface and expedites charge transfer and Na^+^ ions transportation [45,46,47,48,49]. The sodium adsorption capabilities were evaluated through computational models depicting Na^+^ interactions with FeP, Co_2_P, and FeP/Co_2_P heterostructure illustrated in Figure 6f–h. Analyses indicate that the FeP/Co_2_P displays pronounced electronic deficiency compared to its singular counterparts, suggesting that these heterointerfaces are particularly adept at attracting Na^+^ ions, which may enhance the redox reactions. Further insights gleaned from the density of states (DOS) analysis for FeP, Co_2_P, and Fe-Co-P shown in Figure 6i–k indicate that the three materials exhibit overlapping bands at the Fermi level, indicative of metallic properties within their band structure. The elevated DOS at the Fermi level for Fe-Co-P reflects a great availability of electronic states and correlates with it the most excellent conductivity, which is ascribed to the synergistic effects of the FeP/Co_2_P heterojunction formation.

**Figure 7:**
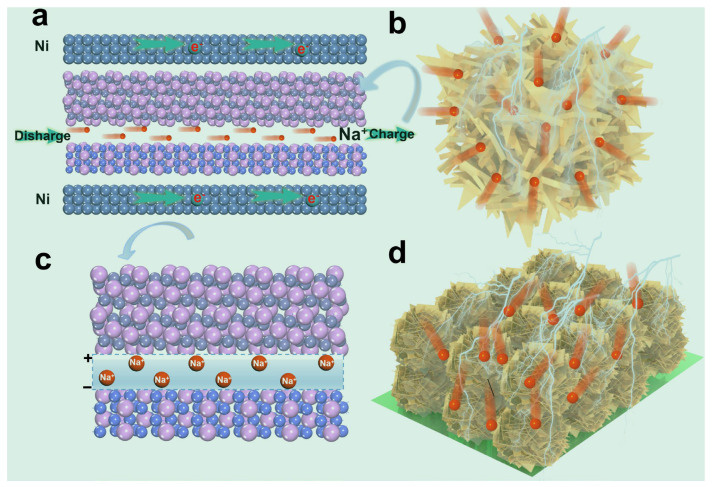
(**a**) Schematic illustration of Fe−Co−P arrays in SIBs. (**b**) Schematic illustration of the three-dimensional Fe−Co−P arrays. (**c**) Schematic illustration of heterostructured Fe−Co−P with inner electric field. (**d**) Schematic illustration of nanosheet-assembled Fe−Co−P microspheres on Ni foam.

In short, the excellent performance of the Fe-Co-P can be summarized in Figure 7. The special self-supported construction with open structure favors fast charge transfer and Na^+^ ion diffusion (Figure 7a). Simultaneously, the abundant voids within the Ni conductive framework and the Fe-Co-P arrays provide sufficient space for the volume change of the host materials (Figure 7b). The nanosheet-assembled Fe-Co-P microspheres occupy maximized active sites for Na^+^ ions and largely favor the infiltration of electrolytes into the inner construction of Fe-Co-P host materials (Figure 7d). Furthermore, the special FeP/Co_2_P heterostructure could considerably mitigate the volume change during repetitive (de)sodiation (Figure 7c). Given the merits of the special Fe-Co-P building, the Fe-Co-P anode delivers satisfactory electrochemical performance in SIBs.

## 4. Conclusions

In summary, the heterostructured Fe-Co-P arrays are rationally constructed in this work, which can be also extended to prepare other metal phosphide arrays. The abundant voids within Ni conductive framework and the Fe-Co-P arrays provide sufficient space for the volume change of FeP and Co_2_P, which is conductive to the thorough infiltration of electrolytes and reduced pathway of Na^+^ ions. As all we know, the conventionally used electrode materials are always mixed with insulated polymer binders to paste on the current collectors, which substantially impedes the charge transfer and deteriorates the electrochemical performance. In contrast, the self-supported arrays can be directly utilized as an electrode with fast charge transfer efficiency. Benefited by the three-dimensional framework of Ni foam, the volume change of Fe-Co-P can be largely mitigated. Furthermore, the special FeP/Co_2_P heterostructure could considerably mitigate the volume change during repetitive (de)sodiation. In addition, the special self-supported construction with open structure favors fast charge transfer and Na^+^ ion diffusion. As expected, the heterostructured Fe-Co-P arrays demonstrate satisfactory performance with high reversible capacities of 634 mAh g^−1^ at 0.2 A g^−1^ and 239 mAh g^−1^ after 300 cycles at 1 A g^−1^. What is more, DFT calculations confirm the built-in electric field in the heterointerfaces, which greatly accelerates charge transfer and Na^+^ ion transportation, thereafter bringing about enhanced electrochemical performance. The Fe-Co-P shows the most excellent electronic conductivity based on the theoretical calculations. The methodology and innovative design mentioned in this work pave the way for building high energy density energy storage devices.

## Data Availability

The data presented in this study are available on reasonable request from the corresponding author.
